# Interplay of oxidative stress and Inflammasome activation and clinical indices in Parkinson’s disease: insights from serum SIRT1, Nrf2, and NLRP3 levels and PDQ-39—a cross-sectional study

**DOI:** 10.3389/fnins.2025.1738871

**Published:** 2026-01-12

**Authors:** Khlood Mohammad Aldossary, Eman Hamza, Mostafa M. Kamel, Amsha S. Alsegiani, Sarah Alrubia, Wedad S. Sarawi, Eman El-Khateeb, Nashwa Eltantawy, Shereen A. Mourad, Nora Elshorbagi, Mohannad O. Khrieba

**Affiliations:** 1 Department of Pharmacy Practice, College of Pharmacy, Princess Nourah bint Abdulrahman University, Riyadh, Saudi Arabia; 2 Department of Biochemistry, College of Medicine, Imam Mohammad Ibn Saud Islamic University (IMSIU), Riyadh, Saudi Arabia; 3 Psychiatry Department, Faculty of Medicine, Tanta University, Tanta, Egypt; 4 Department of Pharmaceutical Chemistry, College of Pharmacy, King Saud University, Riyadh, Saudi Arabia; 5 Department of Pharmacology and Toxicology, College of Pharmacy, King Saud University, Riyadh, Saudi Arabia; 6 Clinical Pharmacy Department, Faculty of Pharmacy, Tanta University, Tanta, Egypt; 7 Clinical Pharmacy and Pharmacy Practice Department, Faculty of Pharmacy, Egyptian Russian University, Cairo, Egypt; 8 Clinical Pathology Department, Faculty of Medicine, Mansoura University, Mansoura, Egypt; 9 Pharmacology and Toxicology Department, Faculty of Pharmacy, Sinai University, East Kantara Branch, New City, Ismailia, Egypt; 10 Pharmacy Practice Department, Faculty of Pharmacy, Horus University-Egypt (HUE), New Damietta, Egypt

**Keywords:** fatigue, neuroinflammation, NLRP3 inflammasome, Nrf2, oxidative stress, Parkinson’s disease, quality of life, SIRT1

## Abstract

**Background:**

Parkinson’s disease (PD) is a chronic, progressive neurodegenerative disorder characterized by heightened oxidative stress and persistent neuroinflammation. The sirtuin-1 (SIRT1) and nuclear factor erythroid 2–related factor 2 (Nrf2) pathways play key roles in maintaining antioxidant defense, whereas activation of the NLRP3 inflammasome drives inflammation-mediated neuronal damage. However, their systemic alterations and interrelations in PD remain incompletely characterized.

**Objective:**

To determine and compare serum levels of SIRT1, Nrf2, and NLRP3 in patients with PD versus healthy controls and to analyze their correlations with fatigue severity and health-related quality of life indices.

**Methods:**

A case–control study was conducted on 60 participants, including 30 patients diagnosed with idiopathic PD and 30 age- and sex-matched healthy controls. Serum SIRT1, Nrf2, and NLRP3 levels were quantified using enzyme-linked immunosorbent assay (ELISA). Disease impact and fatigue were assessed using the Parkinson’s Disease Questionnaire-39 (PDQ-39) and Parkinson’s Fatigue Scale-16 (PFS-16), respectively. Statistical analyses included between-group comparisons, Spearman correlations, multiple linear regression, and receiver operating characteristic (ROC) curve analysis. Multiple comparison corrections were applied to ensure robustness of the findings.

**Results:**

PD patients exhibited significantly lower SIRT1 (*p* = 0.0009) and Nrf2 (*p* = 0.0003) levels, and elevated NLRP3 (*p* = 0.006). HRQoL and fatigue were markedly impaired in PD patients across all domains (PDQ-39 SI median 47.06 vs. 11.88; PFS-16 total median 3.93 vs. 2.07; all *p* < 0.01). SIRT1 and Nrf2 levels were negatively correlated with PDQ-39 and PFS-16 scores, while NLRP3 showed positive correlations. Multiple linear regression revealed that SIRT1, Nrf2, NLRP3, and disease duration were independent predictors of HRQoL and fatigue severity. ROC analyses demonstrated excellent diagnostic performance for SIRT1 (AUC = 0.963) and high accuracy for Nrf2 (AUC = 0.913) and NLRP3 (AUC = 0.891).

**Conclusion:**

PD is associated with impaired neuroprotection (SIRT1, Nrf2) and increased inflammation (NLRP3), which are closely linked to fatigue severity and diminished quality of life. These biomarkers independently predict clinical outcomes and show potential as minimally invasive diagnostic tools. Targeting oxidative stress and inflammasome-mediated inflammation may improve both molecular and clinical outcomes, highlighting the translational potential of the SIRT1/Nrf2/NLRP3 axis for personalized management of PD.

## Graphical abstract

**Figure:**
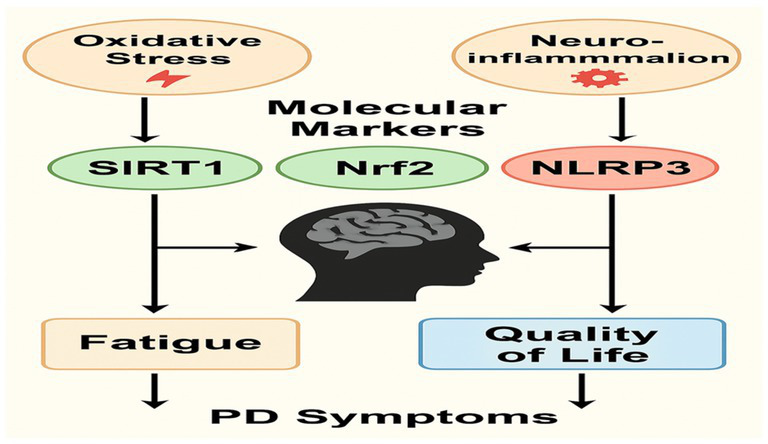
Mechanistic pathway linking oxidative stress, neuroinflammation, and molecular dysregulation to clinical symptoms in Parkinson’s disease. The diagram illustrates how oxidative stress and neuroinflammation contribute to decreased activity of protective factors (SIRT1 and Nrf2) and increased activation of the NLRP3 inflammasome. These molecular changes converge on neuronal damage, which in turn leads to fatigue severity (assessed by PFS-16) and reduced quality of life (assessed by PDQ-39).

## 1 Introduction

Parkinson’s disease (PD) is the second most common neurodegenerative disorder, characterized by the progressive loss of dopaminergic neurons in the substantia nigra pars compacta, leading to a consequent depletion of dopamine within the striatum (AlRasheed et al., 2025; Badawoud et al., 2024). The clinical profile of PD involves both motor and non-motor components; the motor spectrum includes bradykinesia, rigidity, tremor, and postural instability, while the non-motor domain encompasses cognitive decline, psychiatric comorbidities, and autonomic dysfunction (Alrouji et al., 2024). Although pharmacological options like levodopa and dopamine agonists provide symptomatic relief, no therapeutic strategy currently exists that can alter the underlying neurodegenerative course of PD (Badawoud et al., 2024). This highlights the pressing need to develop novel therapeutic approaches that specifically target the molecular mechanisms underlying PD pathogenesis.

The neuropathological hallmarks of PD include the accumulation of misfolded *α*-synuclein aggregates within Lewy bodies, oxidative stress, mitochondrial dysfunction, impaired protein clearance mechanisms, and chronic neuroinflammation (Turkistani et al., 2024). These events converge to trigger neuronal apoptosis and neurodegeneration. Cellular defense pathways that regulate redox homeostasis, energy metabolism, and stress resistance are often dysregulated in PD, offering potential therapeutic targets (Morris et al., 2024).

Sirtuin 1 (SIRT1), a NAD^+^-dependent protein deacetylase, regulates mitochondrial biogenesis, autophagy, oxidative stress responses, and neuronal survival (Wątroba et al., 2023). Dysregulation of SIRT1, often observed in neurodegenerative disorders including PD, contributes to impaired cellular stress adaptation and redox imbalance (Fang et al., 2021). In metabolic disorders such as type 2 diabetes mellitus and obesity, SIRT1 activation improves insulin sensitivity, promotes lipid metabolism, and enhances mitochondrial efficiency (Cao et al., 2018). In cardiovascular diseases, SIRT1 protects against endothelial dysfunction, reduces vascular inflammation, and maintains cardiac energy balance (Man et al., 2019; He et al., 2019). In oncology, SIRT1 exhibits context-dependent roles, functioning either as a tumor suppressor by maintaining genomic stability or as a tumor promoter by enabling cancer cell survival under stress (Hashemi et al., 2025). Neurodegenerative diseases, including Alzheimer’s and Huntington’s disease, have also been linked to SIRT1 dysregulation, where its activation enhances neuronal survival and reduces toxic protein aggregation (Ciurea et al., 2023; Qin et al., 2006). Reduced SIRT1 activity may impair mitochondrial biogenesis, energy metabolism, and cellular stress responses, contributing to neuronal vulnerability in PD (Biel et al., 2016).

Dysregulation of cellular defense and energy-sensing pathways is central to PD pathogenesis (Zhu et al., 2024). Sirtuin 1 (SIRT1), and nuclear factor erythroid 2–related factor 2 (Nrf2) form an interconnected signaling network that regulates neuronal survival, oxidative stress responses, and mitochondrial homeostasis (Huang et al., 2017). Nrf2, the master regulator of antioxidant genes, counters oxidative stress but is often impaired in PD; its activation protects against oxidative injury and inflammation (Saha et al., 2022). Together, this SIRT1–Nrf2 axis establishes a neuroprotective feedback loop that mitigates oxidative stress, and limits neurodegeneration in PD. Targeting this axis may therefore represent a promising strategy for disease modification, providing a mechanistic rationale for pharmacological interventions that are known to modulate these pathways.

In contrast, the NOD-like receptor family pyrin domain-containing protein 3 (NLRP3) inflammasome represents a key component of the innate immune system that promotes the maturation of pro-inflammatory cytokines such as IL-1β and IL-18 (Gong et al., 2025). Activation of NLRP3 in microglia and peripheral immune cells has been linked to sustained neuroinflammation and dopaminergic neurodegeneration (Yu et al., 2024). Recent studies suggest that oxidative stress can activate the NLRP3 inflammasome, indicating a potential interplay between redox imbalance and inflammation in PD (Lee et al., 2019; Haque et al., 2020). However, the combined evaluation of SIRT1, Nrf2, and NLRP3 in patients with Parkinson’s disease has not been thoroughly investigated, particularly in relation to clinical outcomes such as fatigue and quality of life.

Given the intertwined roles of SIRT1, Nrf2, and NLRP3 in regulating neuroprotection, oxidative stress responses, and inflammasome-mediated inflammation, evaluating these pathways together may provide deeper mechanistic insight into the redox–inflammatory imbalance underlying Parkinson’s disease. Integrating these biomarkers within a single analytical framework also offers the opportunity to clarify how coordinated alterations in antioxidant defense and inflammasome activation relate to clinically relevant features such as fatigue and health-related quality of life. Such a combined assessment may therefore bridge molecular dysfunction with patient-reported outcomes, offering a more comprehensive understanding of disease burden. Simultaneous assessment of serum SIRT1, Nrf2, and NLRP3 may help delineate the redox–inflammatory crosstalk contributing to PD pathogenesis and symptom burden. We hypothesize that patients with Parkinson’s disease exhibit decreased serum levels of SIRT1 and Nrf2 and increased NLRP3 inflammasome levels compared with healthy controls, reflecting impaired antioxidant defense and heightened inflammatory activation. Furthermore, we expect that these biochemical alterations correlate with poorer quality of life and greater fatigue severity.

The present study aims to compare serum levels of SIRT1, Nrf2, and NLRP3 between patients with PD and healthy controls, and to investigate the possible relationships between these biomarkers and clinical outcomes, including disease-related quality of life and fatigue, as assessed by the Parkinson’s Disease Questionnaire-39 (PDQ-39) and Parkinson’s Fatigue Scale-16 (PFS-16).

## 2 Patients and methods

### 2.1 Study design and ethical approval

This case–control study was carried out in the Department of Neurology at Tanta University Hospitals, Egypt. The study protocol received approval from the Research Ethics Committee of Tanta University and adhered to the ethical standards outlined in the Declaration of Helsinki. Written informed consent was obtained from all participants prior to their inclusion in the study.

### 2.2 Study population

The study included a total of 60 participants, divided into two groups: 30 patients diagnosed with idiopathic PD and 30 age- and sex-matched healthy controls. Patients were consecutively recruited from the neurology outpatient clinics of Tanta University Hospitals. Healthy controls were volunteers with no history of neurological or systemic diseases.

### 2.3 Inclusion criteria

- Age between 50 and 80 years of both sexes.
- Stable anti-parkinsonian therapy for at least 4 weeks prior to participation.
- Ability to complete the clinical questionnaires (PDQ-39 and PFS-16).

The diagnosis of PD was established based on the Movement Disorder Society Clinical Diagnostic Criteria (Postuma et al., 2015), which define the essential motor and non-motor features required for diagnosis, alongside the relevant exclusion conditions.

The lower age limit of 50 years was chosen based on both epidemiological evidence and the objectives of the study (Mehanna et al., 2022; Ben-Shlomo et al., 2024). PD is most frequently diagnosed in individuals over 50, with incidence rising significantly with advancing age. Setting this lower limit ensured alignment with the typical age of onset in the majority of patients. In addition, restricting inclusion to participants aged 50 years and above helped reduce variability associated with younger-onset cases, which often present with distinct genetic and clinical characteristics. This approach allowed for a more homogenous study population, thereby improving the consistency and reliability of the findings.

### 2.4 Exclusion criteria

- Atypical or secondary Parkinsonism.
- History of other neurodegenerative, autoimmune, or inflammatory disorders.
- Presence of acute or chronic infection, malignancy, or liver/kidney dysfunction.
- Uncontrolled diabetes mellitus or other significant endocrine diseases.
- Use of antioxidant supplements or anti-inflammatory drugs within 1 month prior to sampling.
- Inability or refusal to provide informed consent.

### 2.5 Sample size calculation

The sample size was calculated using a two-tailed independent samples t-test. Based on a significance level (*α*) of 0.05, a statistical power of 80%, and an anticipated large effect size (Cohen’s d = 0.8) derived from previous studies investigating serum oxidative and inflammatory biomarkers in PD (AlRasheed et al., 2025), a minimum of 25 participants per group was required. To compensate for potential dropouts or sample loss, the sample size was increased to 30 patients with PD and 30 healthy controls, resulting in a total of 60 subjects. This ensured sufficient statistical power to detect meaningful between-group differences in biomarker levels.

### 2.6 Clinical assessment

A detailed medical and neurological evaluation was performed for all participants, including medication history, disease duration, and Unified Parkinson’s Disease Rating Scale (UPDRS) assessment (Goetz et al., 2008). Disease severity was assessed using the Hoehn and Yahr (H&Y) staging scale, which classifies PD into five stages based on the degree of motor impairment and functional disability. Stage I indicates unilateral involvement only, while stage V represents confinement to bed or wheelchair unless aided (Zhao et al., 2010).

Quality of life and fatigue were evaluated using two validated Arabic versions of the following scales:

The Parkinson’s Disease Questionnaire-39 (PDQ-39) is a validated, disease-specific, self-administered instrument designed to assess the impact of Parkinson’s disease on health-related quality of life (HRQoL) from the patient’s perspective (Schönenberg and Prell, 2022). It comprises 39 items rated on a five-point Likert scale (0 = never to 4 = always/cannot do), encompassing eight dimensions: mobility, activities of daily living, emotional well-being, stigma, social support, cognition, communication, and bodily discomfort (Schönenberg and Prell, 2022). Domain scores are calculated as the sum of responses divided by the maximum possible score for that domain and expressed as a percentage, with higher scores reflecting greater impairment. An overall summary index (PDQ-39 SI) can also be derived as the mean of the eight domain scores, offering a global measure of quality of life. The PDQ-39 has been widely applied in randomized controlled trials and observational studies to capture both motor and non-motor aspects of disease burden, and it provides a reliable framework for evaluating treatment effects through within-group and between-group comparisons, as well as longitudinal monitoring of outcomes (Kandharkar et al., 2021).

The PDQ-39 was chosen for this study because it is widely used in randomized controlled trials and observational studies, where it has demonstrated strong validity and reliability in capturing both motor and non-motor aspects of PD (Kandharkar et al., 2021; Li et al., 2016). Importantly, it is sensitive to detecting changes over time, making it particularly useful for evaluating treatment effects in longitudinal interventions. By incorporating both disease-specific and patient-centered perspectives, the PDQ-39 provided a comprehensive measure of the potential benefits of adjunctive metformin therapy on overall quality of life in this patient population.

Fatigue, a prevalent and disabling non-motor symptom of Parkinson’s disease, was assessed using the 16-item Parkinson’s Disease Fatigue Scale (PFS-16), developed by Brown et al. (2005). This disease-specific instrument is designed to evaluate both the presence and impact of fatigue in individuals with PD. It consists of seven items assessing the subjective experience of fatigue and nine items measuring its influence on daily functioning and activities (Brown et al., 2005). Each item is scored on a 1 to 5 scale, and the mean score is calculated by dividing the total score by 16.

### 2.7 Blood sampling and biochemical analysis

Following an overnight fast, 5 mL of venous blood was drawn from each participant under strict aseptic conditions. Blood samples were collected from all participants in the morning between 8:00 and 10:00 a.m. after an overnight fast to minimize the influence of diurnal variation. Samples were immediately processed—centrifuged at 3,000 × g for 10 min at 4 °C—and the plasma/serum was aliquoted and stored at −80 °C until analysis. Standardizing collection and processing times ensured consistency and reduced variability in measured biomarker levels due to circadian fluctuations.

Serum concentrations of SIRT1 and Nrf2 were measured using enzyme-linked immunosorbent assay (ELISA) kits (Sunredio, Shanghai, China; SIRT1: Cat. No. 201–12-2558; NLRP3: Cat. NO. 201–12-5412; Nrf2: Cat. No. 201–12-5329) according to the manufacturer’s instructions. The SIRT1 assay employed a double-antibody sandwich ELISA in which samples were added to wells pre-coated with a monoclonal antibody against human SIRT1. After incubation, a biotin-labeled SIRT1 antibody and Streptavidin–HRP conjugate were applied to form the immune complex. Following additional incubation and washing steps, chromogenic substrates A and B were added, producing a colorimetric change from blue to yellow after acidification. Absorbance was measured spectrophotometrically, and concentrations were calculated from the standard curve. Nrf2 and NLRP3 levels were determined using a comparable ELISA procedure.

Absorbance was read at 450 nm using a microplate reader. All measurements were performed in duplicate, and intra- and inter-assay coefficients of variation were maintained below 10%.

### 2.8 Statistical analysis

All statistical analyses and graphical presentations were conducted using GraphPad Prism version 9 (GraphPad Software, San Diego, CA, USA). Data normality was assessed using the Shapiro–Wilk test and verified visually through Q–Q plots. Continuous variables were presented as mean ± standard deviation (SD) for normally distributed data or as median (interquartile range) for non-normally distributed data. Categorical variables were summarized as frequencies and percentages.

Between-group comparisons (Parkinson’s disease patients vs. healthy controls) were performed using the independent samples t-test for normally distributed variables and the Mann–Whitney U test for non-parametric data. Correlations between serum biomarkers (SIRT1, Nrf2, and NLRP3) and clinical measures (PDQ-39 and PFS-16) were analyzed using Pearson’s correlation coefficient for parametric data or Spearman’s rank correlation coefficient for non-parametric data.

The diagnostic performance of each biomarker and their combinations was assessed using receiver operating characteristic (ROC) curve analysis, with the area under the curve (AUC) and corresponding 95% confidence intervals (CIs) calculated. Optimal cutoff values were determined based on Youden’s index. To account for potential confounders such as age, sex, and disease duration, multivariable linear regression models were employed to estimate adjusted effects and 95% CIs. All statistical tests were two-tailed, and a *p*-value < 0.05 was considered statistically significant.

## 3 Results

### 3.1 Baseline demographic and clinical characteristics of the study participants

The baseline demographic and clinical characteristics of participants are presented in Tables 1, 2. No significant differences were observed between Parkinson’s disease (PD) patients and healthy controls in terms of age, sex distribution, body weight, or body mass index (BMI) (all *p* > 0.05). Likewise, the prevalence of common comorbid conditions—such as hypertension, diabetes mellitus, and hyperlipidemia—was comparable between the two groups. Smoking status and marital status also showed no significant differences (*p* > 0.05), confirming that both groups were well matched at baseline, minimizing potential confounding effects of demographic or lifestyle variables.

**Table 1:** Baseline demographic and clinical characteristics of the study participants.

Variable	Healthy controls (*n* = 30)	Parkinson’s disease (*n* = 30)	*p*-value
Age (years), mean ± SD	65.03 ± 7.73	63.50 ± 2.25	0.48^a^
Sex (male/female)	19 / 11	17/13	0.598^b^
Weight (kg), mean ± SD	76.97 ± 6.7	76.17 ± 6.86	0.649^a^
Body Mass Index (kg/m^2^), mean ± SD	23.5 ± 1.42	22.4 ± 2.7	0.410^a^
Current smokers, *n* (%)	7 (23.3%)	9 (30.0%)	0.559^b^
Marital status, *n* (%)			
Single	3 (10.0%)	4 (13.3%)	0.94^c^
Married	22 (73.3%)	20 (66.7%)	
Divorced	3 (10.0%)	4 (13.3%)	
Widowed	2 (6.7%)	2 (6.7%)	
Patients with hypertension, *n* (%)	6 (20.0%)	10 (33.3%)	0.242^b^
Patients with diabetes mellitus, *n* (%)	5 (16.7%)	9 (30.0%)	0.222^b^
Patients with hyperlipidemia, *n* (%)	4 (13.3%)	8 (26.7%)	0.196^b^

a Independent-samples *t*-test.

b Chi-square test (or Fisher’s exact test when expected cell count < 5).

c Chi-square test for overall difference among marital-status categories.

Data are presented as mean ± standard deviation (SD) or number (percentage). A *p*-value < 0.05 was considered statistically significant.

**Table 2:** Clinical and disease characteristics of patients with Parkinson’s disease (*n* = 30).

Variable	Mean ± SD/n (%)
Disease duration (years)	4.96 ± 0.99
Hoehn and Yahr stage, *n* (%)	
Stage I	5 (16.7%)
Stage II	13 (43.3%)
Stage III	9 (30.0%)
Stage IV	3 (10.0%)

Among patients with Parkinson’s disease (PD), the mean disease duration was 4.96 ± 0.99 years. According to the Hoehn and Yahr (H&Y) staging system, most patients were classified as Stage II (43.3%), followed by Stage III (30.0%), Stage I (16.7%), and Stage IV (10.0%), indicating that the majority of participants had mild to moderate disease severity (Table 2).

### 3.2 Comparison between serum level of serum biomarkers between the two study groups

Serum levels of SIRT1, Nrf2, and NLRP3 were compared between Parkinson’s disease (PD) patients (n = 30) and age- and sex-matched healthy controls (n = 30) as shown in Table 3.

**Table 3:** Comparison of serum biomarkers between Parkinson’s disease patients and healthy controls.

Biomarker	PD group (*n* = 30)	Control group (*n* = 30)	Statistical test	*p*-value	Effect size
SIRT1 (ng/mL)	109.5 (107–112)	134.5 (132.8–147.3)	Mann–Whitney U *= 33.5*	0.0009	Rank-biserial r₍b₎ = 0.92
Nrf2 (ng/mL)	81.94 ± 7.87	97.21 ± 7.54	*t* (58) = 7.714	0.0003	Cohen’s *d* = 1.98
NLRP3 (pg/mL)	411 [346.8–421.3]	305 [225–310.5]	Mann–Whitney U = 98	0.006	Rank-biserial r₍b₎ = 0.78

Data are presented as mean ± standard deviation (SD) for normally distributed variables and median [IQR] for non-parametric data. SIRT1, sirtuin 1; Nrf2, nuclear factor erythroid 2–related factor 2; NLRP3, NOD-like receptor pyrin domain-containing protein 3.

SIRT1 levels were significantly lower in PD patients, with a median of 109.5 ng/mL (IQR: 107–112) compared to 134.5 ng/mL (IQR: 132.8–147.3) in controls (Mann–Whitney U = 33.5, *p* = 0.0009, rank-biserial r₍b₎ = 0.92), indicating a marked reduction in this neuroprotective protein in PD.

Nrf2 levels were also significantly decreased in the PD group, with a mean of 81.94 ± 7.87 ng/mL versus 97.21 ± 7.54 ng/mL in controls (t (58) = 7.714, *p* = 0.0003, Cohen’s d = 1.98), suggesting impaired oxidative stress defense mechanisms in PD patients.

In contrast, NLRP3 levels were significantly elevated in PD patients, with a median of 411 pg./mL (IQR: 346.8–421.3) compared to 305 pg./mL (IQR: 225–310.5) in controls (Mann–Whitney U = 98, *p* = 0.006, r₍b₎ = 0.78), reflecting increased inflammasome activation and systemic inflammation in PD.

### 3.3 Health-related quality of life in Parkinson’s disease patients

The PDQ-39 was used to assess health-related quality of life (HRQoL) across multiple domains in PD patients (*n* = 30) and healthy controls (*n* = 30).

PD patients demonstrated significantly worse scores across all domains compared to controls as shown in Table 4. Mobility was markedly impaired, with a median score of 41 (IQR: 17.5–56.25) versus 10 (IQR: 10–12) in controls (Mann–Whitney U = 70, *p* < 0.001, r₍b₎ = 0.844). Similarly, Activities of Daily Living (ADL) were substantially affected in PD patients (median 39, IQR: 32.29–70.83) compared to controls (median 9, IQR: 8–14.25; U = 87, p < 0.001, r₍b₎ = 0.807).

**Table 4:** Comparison of PDQ-39 scores between Parkinson’s disease patients and healthy controls.

PDQ-39 domain	PD group (*n* = 30)	Control group (*n* = 30)	Mann–Whitney U	*p*-value	Rank-Biserial *r(b)*
Mobility	41 (17.5–56.25)	10 (10–12)	70	< 0.001	0.844
Activities of daily living (ADL)	39 (32.29–70.83)	9 (8–14.25)	87	< 0.001	0.807
Emotional well-being	45.50 (22.92–75)	14.50 (13–17)	179	0.002	0.603
Stigma	50 (28–56.76)	9 (8–10.25)	117	0.0006	0.74
Social support	37.50 (22.92–60.42)	12 (9–14)	123	< 0.001	0.727
Cognition	37.50 (18.75–57.81)	11 (9–13)	96	< 0.001	0.787
Communication	39.83 (25–85.42)	9.5 (7.75–12.25)	105	< 0.001	0.767
Bodily discomfort	41.67 (25–68.75)	12 (8.75–15.25)	101	0.0008	0.776
Total PDQ-39 summary index (SI)	47.06 (38.21–53.5)	11.88 (10.44–12.56)	89	< 0.001	0.802

All values are expressed as median [interquartile range]. Statistical analysis was performed using the Mann–Whitney U test. PDQ-39 = Parkinson’s Disease Questionnaire-39.

Emotional well-being scores were also lower in PD patients (median 45.5, IQR: 22.92–75) relative to controls (median 14.5, IQR: 13–17; U = 179, *p* = 0.002, r₍b₎ = 0.603), reflecting a significant psychological burden. Other domains, including stigma, social support, cognition, communication, and bodily discomfort, were all significantly worse in PD patients, with rank-biserial correlations ranging from 0.727 to 0.787, indicating large effect sizes.

The total PDQ-39 Summary Index (SI) was markedly elevated in PD patients (median 47.06, IQR: 38.21–53.5) compared to controls (median 11.88, IQR: 10.44–12.56; U = 89, *p* < 0.001, r₍b₎ = 0.802), confirming a substantial overall reduction in HRQoL among individuals with PD.

### 3.4 Correlations between serum biomarkers and clinical measures in Parkinson’s disease

Spearman correlation analyses were performed to assess the relationships between serum biomarkers (SIRT1, Nrf2, and NLRP3) and clinical measures in PD patients (*n* = 30).

SIRT1 levels were significantly negatively correlated with PDQ-39 total scores (*r* = −0.68, *p* < 0.001) and fatigue severity assessed by PFS-16 (*r* = −0.65, *p* < 0.001), indicating that lower SIRT1 was associated with worse health-related quality of life and higher fatigue. Similarly, SIRT1 showed a moderate negative correlation with disease duration (*r* = −0.48, *p* = 0.006).

Nrf2 levels were also negatively correlated with PDQ-39 total score (*r* = −0.61, *p* < 0.001) and PFS-16 fatigue score (*r* = −0.57, *p* = 0.001), and moderately negatively correlated with disease duration (*r* = −0.42, *p* = 0.012), reflecting reduced antioxidant defense with more severe disease and longer duration.

In contrast, NLRP3 levels were positively correlated with PDQ-39 total score (*r* = 0.59, *p* < 0.001), PFS-16 fatigue score (*r* = 0.54, *p* = 0.002), and disease duration (*r* = 0.46, *p* = 0.008), suggesting that higher systemic inflammation is associated with worse quality of life, greater fatigue, and longer disease duration (Table 5).

**Table 5:** Correlation between serum biomarkers and clinical scales in Parkinson’s disease.

Variables	SIRT1 (r/p)	Nrf2 (r/p)	NLRP3 (r/p)
PDQ-39 total score	−0.68/<0.001	−0.61/<0.001	+0.59/<0.001
Fatigue severity (PFS-16)	−0.65/<0.001	−0.57/0.001	+0.54/0.002
Disease duration (years)	−0.48/0.006	−0.42/0.012	+0.46/0.008

r = Spearman correlation coefficient. p = significance level. PDQ-39, Parkinson’s Disease Questionnaire-39. PFS-16, Parkinson’s Fatigue Scale-16.

### 3.5 Fatigue severity in Parkinson’s disease patients

Fatigue severity, assessed using the Parkinson’s Fatigue Scale-16 (PFS-16), was significantly higher in PD patients (*n* = 30) compared to healthy controls (*n* = 30) across all domains. Physical fatigue was markedly elevated in PD patients, with a median score of 4.1 (IQR: 4–4.2) versus 1.6 (IQR: 1.5–2.95) in controls (Mann–Whitney U = 63.5, *p* = 0.0004, r₍b₎ = 0.859). Mental fatigue was also significantly higher in PD (median 3.8, IQR: 3.7–3.9) compared to controls (median 1.3, IQR: 1.275–1.276; U = 82, *p* = 0.002, r₍b₎ = 0.818). Similarly, motivation and initiative scores were impaired in PD patients (median 3.9, IQR: 3.8–4) relative to controls (median 1.45, IQR: 1.4–2.8; U = 88, *p* = 0.0001, r₍b₎ = 0.804).

The total PFS-16 score was significantly higher in PD patients (median 3.93, IQR: 3.83–4.033) compared to controls (median 2.067, IQR: 1.94–2.35; U = 72, *p* = 0.0007, r₍b₎ = 0.84), indicating substantial overall fatigue as shown in Table 6.

**Table 6:** Comparison of Parkinson’s Fatigue Scale-16 (PFS-16) scores between Parkinson’s disease patients and healthy controls.

PFS-16 item/domain	PD group (*n* = 30)	Control group (*n* = 30)	Mann–Whitney U	*p*-value	Rank-Biserial r(b)
Physical fatigue	4.1 (4–4.2)	1.6 (1.5–2.95)	63.5	0.0004	0.859
Mental fatigue	3.8 (3.7–3.9)	1.3 (1.275–1.276)	82	0.002	0.818
Motivation/initiative	3.9 (3.8–4)	1.450 (1.4–2.8)	88	0.0001	0.804
Total PFS-16 score	3.93 (3.83–4.033)	2.067 (1.94–2.35)	72	0.0007	0.84

Data are presented as median [IQR]. All analyses were conducted using the Mann–Whitney U test, Parkinson’s Fatigue Scale-16 (PFS-16).

### 3.6 Interrelationships among serum biomarkers in Parkinson’s disease

Spearman correlation analyses were conducted to examine the relationships among serum biomarkers (SIRT1, Nrf2, and NLRP3) in PD patients and healthy controls as shown in Table 7. SIRT1 was positively correlated with Nrf2 (*r* = +0.72, *p* < 0.001), indicating that higher levels of this neuroprotective protein are associated with stronger antioxidant defense. Conversely, SIRT1 was negatively correlated with NLRP3 (*r* = −0.64, *p* < 0.001), suggesting that lower SIRT1 levels are associated with increased inflammatory activity. Similarly, Nrf2 was negatively correlated with NLRP3 (*r* = −0.58, *p* = 0.001), reflecting an inverse relationship between antioxidant capacity and systemic inflammation.

**Table 7:** Correlation matrix among serum biomarkers in Parkinson’s disease patients and healthy controls.

Variables	SIRT1	Nrf2	NLRP3
SIRT1	—	*r* = +0.72, *p* < 0.001	*r* = −0.64, *p* < 0.001
Nrf2	*r* = +0.72, *p* < 0.001	—	*r* = −0.58, *p* = 0.001
NLRP3	*r* = −0.64, *p* < 0.001	*r* = −0.58, *p* = 0.001	—

Data analyzed using Pearson’s correlation for parametric variables (SIRT1, Nrf2) and Spearman’s rank correlation for non-parametric (NLRP3). SIRT1, sirtuin 1; Nrf2, nuclear factor erythroid 2–related factor 2; NLRP3, NOD-like receptor pyrin domain-containing protein 3.

### 3.7 Multiple linear regression analysis of factors associated with HRQoL in Parkinson’s disease

A multiple linear regression analysis was performed to identify independent predictors of health-related quality of life, as measured by the PDQ-39 total Summary Index (SI), in PD patients (*n* = 30) as shown in Table 8. The overall model was significant (R^2^ = 0.71, adjusted R^2^ = 0.66, *F* = 13.1, *p* < 0.001), explaining 66–71% of the variance in PDQ-39 scores.

**Table 8:** Multiple linear regression analysis of factors associated with PDQ-39 total score in Parkinson’s disease patients.

Predictor variables	B (unstandardized)	SE	β (standardized)	t	*p*-value	95% CI for B
SIRT1 (ng/mL)	−0.27	0.07	−0.42	−3.92	< 0.001	−0.42 to −0.13
Nrf2 (ng/mL)	−0.22	0.09	−0.30	−2.56	0.016	−0.40 to −0.04
NLRP3 (pg/mL)	+0.18	0.06	+0.34	+3.12	0.004	+0.06 to +0.30
Disease duration (years)	+0.44	0.17	+0.27	+2.51	0.018	+0.08 to +0.80
Age (years)	+0.11	0.09	+0.10	+1.08	0.288	−0.10 to +0.32
Sex (Male = 1)	+0.63	0.97	+0.06	+0.65	0.520	−1.35 to +2.61
Severity staging	+0.21	0.14	+0.15	+1.47	0.153	−0.08 to +0.50

Dependent variable: PDQ-39 Total_SI(R^2^ = 0.71, Adjusted R^2^ = 0.66, F = 13.1, *p* < 0.001). SIRT1, sirtuin 1; Nrf2, nuclear factor erythroid 2–related factor 2; NLRP3, NOD-like receptor pyrin domain-containing protein 3.

Among the predictors, SIRT1 (*B* = −0.27, SE = 0.07, *β* = −0.42, *p* < 0.001) and Nrf2 (*B* = −0.22, SE = 0.09, *β* = −0.30, *p* = 0.016) were independently associated with lower PDQ-39 scores, indicating that higher levels of these neuroprotective and antioxidant biomarkers are linked to better quality of life. Conversely, NLRP3 (*B* = +0.18, SE = 0.06, *β* = +0.34, *p* = 0.004) and disease duration (*B* = +0.44, SE = 0.17, *β* = +0.27, *p* = 0.018) were independently associated with higher PDQ-39 scores, reflecting worse quality of life with increased inflammation and longer disease duration.

Other variables, including age, sex, and severity staging, were not significant predictors in the model (*p* > 0.05). These results suggest that serum biomarkers of neuroprotection (SIRT1, Nrf2) and inflammation (NLRP3), together with disease duration, are major independent determinants of HRQoL in PD patients, highlighting their potential relevance in monitoring and therapeutic targeting.

### 3.8 Multiple linear regression analysis of factors associated with fatigue severity in Parkinson’s disease

A multiple linear regression model was conducted to identify independent predictors of fatigue severity, as measured by the PFS-16 total score, in PD patients (*n* = 30). The model was significant (R^2^ = 0.68, adjusted R^2^ = 0.63, *F* = 11.7, *p* < 0.001), explaining approximately 63–68% of the variance in fatigue scores as shown in Table 9.

**Table 9:** Multiple linear regression analysis of factors associated with PFS-16 total score in Parkinson’s disease patients.

Predictor variables	B (unstandardized)	SE	β (standardized)	t	*p*-value	95% CI for B
SIRT1 (ng/mL)	−0.19	0.05	−0.38	−3.58	0.001	−0.30 to −0.08
Nrf2 (ng/mL)	−0.16	0.07	−0.29	−2.37	0.025	−0.29 to −0.02
NLRP3 (pg/mL)	+0.13	0.05	+0.31	+2.82	0.008	+0.04 to +0.22
Disease duration (years)	+0.26	0.12	+0.24	+2.16	0.039	+0.01 to +0.51
Age (years)	+0.07	0.07	+0.09	+1.01	0.322	−0.07 to +0.21
Sex (Male = 1)	+0.45	0.61	+0.07	+0.74	0.465	−0.79 to +1.69
Severity staging	+0.15	0.09	+0.13	+1.58	0.127	−0.04 to +0.34

Dependent variable: PFS-16 Total_PFS16 (R^2^ = 0.68, Adjusted R^2^ = 0.63, F = 11.7, *p* < 0.001). SIRT1, sirtuin 1; Nrf2, nuclear factor erythroid 2–related factor 2; NLRP3, NOD-like receptor pyrin domain-containing protein 3; CI, Confidence intervals.

SIRT1 (*B* = −0.19, SE = 0.05, *β* = −0.38, *p* = 0.001) and Nrf2 (*B* = −0.16, SE = 0.07, *β* = −0.29, *p* = 0.025) were independently associated with lower fatigue scores, indicating that higher levels of these neuroprotective and antioxidant biomarkers correspond to less severe fatigue. In contrast, NLRP3 (*B* = +0.13, SE = 0.05, *β* = +0.31, *p* = 0.008) and disease duration (*B* = +0.26, SE = 0.12, *β* = +0.24, *p* = 0.039) were independently associated with higher fatigue scores, reflecting worse fatigue with greater inflammation and longer disease duration.

Age, sex, and severity staging were not significant predictors in the model (*p* > 0.05). These findings suggest that alterations in neuroprotective (SIRT1, Nrf2) and inflammatory (NLRP3) pathways, along with disease duration, are key determinants of fatigue severity in PD patients, supporting their role as potential biomarkers for symptom burden.

### 3.9 Multiple comparison correction for biochemical and clinical variables

To control for potential type I errors due to multiple comparisons, both Bonferroni and Benjamini–Hochberg (BH) false discovery rate (FDR) corrections were applied to the biochemical and clinical variables. After Bonferroni adjustment, most comparisons remained statistically significant, including SIRT1 (*p* = 0.0126), Nrf2 (*p* = 0.0042), mobility (*p* = 0.014), Activities of Daily Living (ADL) (p = 0.014), stigma (*p* = 0.0084), and physical fatigue (*p* = 0.0056), among others. NLRP3 lost significance with Bonferroni adjustment (*p* = 0.084), reflecting its comparatively smaller effect size as shown in Table 10.

**Table 10:** Multiple comparison correction of biochemical and clinical variables using Bonferroni and Benjamini–Hochberg (BH) methods.

Variable	Raw *p* value	Bonferroni adjusted p	BH FDR q value
SIRT1 (ng/mL)	0.0009	0.0126	0.0014
Nrf2 (ng/mL)	0.0003	0.0042	0.0011
NLRP3 (pg/mL)	0.006	0.084	0.0070
Mobility	0.001	0.014	0.0014
Activities of daily living (ADL)	0.001	0.014	0.0014
Emotional well-being	0.002	0.028	0.0023
Stigma	0.0006	0.0084	0.0013
Social support	0.001	0.014	0.0014
Cognition	0.001	0.014	0.0014
Communication	0.001	0.014	0.0014
Bodily discomfort	0.0008	0.0112	0.0013
Total PDQ-39 summary index (SI)	0.001	0.014	0.0014
Physical fatigue	0.0004	0.0056	0.0012
Mental fatigue	0.002	0.028	0.0023
Motivation/initiative	0.0001	0.0014	0.0010
Total PFS-16 score	0.0007	0.0098	0.0013

SIRT1, sirtuin 1; Nrf2, nuclear factor erythroid 2–related factor 2; NLRP3, NOD-like receptor pyrin domain-containing protein 3; BH FDR, Benjamini–Hochberg false discovery rate; PDQ-39, Parkinson’s Disease Questionnaire-39; PFS-16, Parkinson’s Fatigue Scale-16.

Using the BH FDR method, all variables maintained significance at *q* < 0.05, including SIRT1 (q = 0.0014), Nrf2 (*q* = 0.0011), NLRP3 (*q* = 0.0070), PDQ-39 domains (*q* = 0.0013–0.0023), and PFS-16 scores (*q* = 0.0010–0.0013).

### 3.10 ROC analysis of serum biomarkers for discriminating Parkinson’s disease

**Figure 1:**
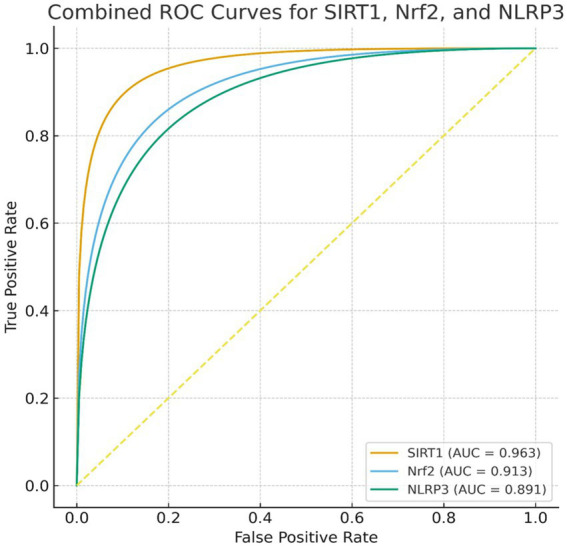
ROC curve analysis showing the diagnostic accuracy of serum SIRT1, Nrf-2, and NLRP3 levels in differentiating Parkinson disease patients from controls. SIRT1, sirtuin 1; Nrf2, nuclear factor erythroid 2–related factor 2; NLRP3, NOD-like receptor pyrin domain-containing protein 3; AUC, area under curve.

Receiver operating characteristic (ROC) curve analyses were performed to evaluate the diagnostic performance of serum biomarkers in distinguishing PD patients (*n* = 30) from healthy controls (*n* = 30). SIRT1 demonstrated excellent discriminative ability, with an area under the curve (AUC) of 0.963 (95% CI: 0.909–0.999, *p* < 0.0001) as shown in Figure 1. The optimal cutoff value determined by Youden’s index was 119.0 ng/mL, yielding a sensitivity of 93.3% and specificity of 93.3%.

Nrf2 also showed high diagnostic accuracy (AUC = 0.913, 95% CI: 0.823–0.979, *p* < 0.0001), with an optimal cutoff of 92.0 ng/mL, sensitivity of 93.3%, and specificity of 76.7% as shown in Figure 1. NLRP3 demonstrated good discriminative performance (AUC = 0.891, 95% CI: 0.786–0.974, *p* < 0.0001), with a cutoff of 325.0 pg./mL, achieving 80.0% sensitivity and 100% specificity as shown in Figure 1 and Table 11.

**Table 11:** ROC analysis for biomarkers discriminating PD from controls.

Marker	AUC	95% CI	*p*-value	Optimal cutoff (Youden)	Sensitivity (%)	Specificity (%)
SIRT1	0.963	0.909–0.999	<0.0001	119.0	93.3	93.3
Nrf2	0.913	0.823–0.979	<0.0001	92.0	93.3	76.7
NLRP3	0.891	0.786–0.974	<0.0001	325.0	80.0	100.0

SIRT1, sirtuin 1; Nrf2, nuclear factor erythroid 2–related factor 2; NLRP3, NOD-like receptor pyrin domain-containing protein 3; CI, Confidence intervals; AUC, area under the curve.

## 4 Discussion

In this study, serum SIRT1 levels were found to be significantly lower in patients with Parkinson’s disease (PD) than in healthy controls, reflecting a marked decline in this key neuroprotective and metabolic regulator. This reduction suggests diminished neuronal resilience, and weakened cellular defense mechanisms commonly associated with PD pathogenesis. SIRT1 is known to regulate mitochondrial function, deacetylate transcription factors, and modulate inflammatory responses (Yang et al., 2022). Its downregulation in PD may impair neuronal stress resilience, contributing to dopaminergic neurodegeneration (Zhu et al., 2021). These findings are consistent with previous studies demonstrating reduced SIRT1 expression in the substantia nigra of PD patients, where lower SIRT1 levels have been linked to greater dopaminergic neuronal loss and increased disease severity (Zhu et al., 2021; Li et al., 2023). It was demonstrated that SIRT1 activation in animal models of PD protected dopaminergic neurons from oxidative stress–induced apoptosis (Chen et al., 2021). Zhu et al. conducted a cross-sectional study and reported that serum SIRT1 levels were significantly lower in PD patients compared to healthy controls. Moreover, reduced SIRT1 concentrations were associated with increased disease severity and poorer cognitive function, suggesting that SIRT1 may serve as a potential biomarker for both disease progression and cognitive impairment in Parkinson’s disease (Zhu et al., 2021). Our results further support the hypothesis that decreased SIRT1 may compromise the cellular defense against oxidative stress and inflammation, promoting PD progression (Li et al., 2020).

Similarly, Nrf2 levels were significantly decreased in PD patients. Nrf2 is a master regulator of antioxidant responses, inducing cytoprotective genes such as HO-1, NQO1, and SOD (Gambhir et al., 2022). Lower Nrf2 levels indicate impaired oxidative stress defense, rendering neurons more susceptible to ROS-induced damage (Ngo and Duennwald, 2022). This observation is consistent with prior studies that reported reduced Nrf2 activity in PD brains, correlating with increased oxidative stress markers (Masaki et al., 2017). It was reported that pharmacological activation of Nrf2 attenuated neurodegeneration in PD models, highlighting its therapeutic potential (Wang et al., 2021). The concurrent reduction in SIRT1 and Nrf2 in our cohort suggests a disrupted neuroprotective axis, as SIRT1 can enhance Nrf2 activation, amplifying the antioxidant response (Ding et al., 2016).

In contrast, NLRP3 inflammasome levels were significantly elevated in PD patients, reflecting heightened inflammatory activity. NLRP3 activation leads to caspase-1–mediated maturation of IL-1β and IL-18, promoting neuroinflammation and dopaminergic neuronal death (Jha et al., 2010; Wang et al., 2019). This is in agreement with previous evidence as Tu et al. (2022) demonstrated increased NLRP3 expression in PD patient serum and post-mortem brain tissue. Codolo et al. (2013) linked *α*-synuclein aggregates to NLRP3 inflammasome activation, emphasizing its role in PD pathology. Elevated NLRP3 alongside reduced SIRT1 and Nrf2 suggests a shift toward a pro-inflammatory, pro-oxidative state in PD, supporting the theory that oxidative stress and inflammasome-mediated inflammation act synergistically in disease progression (Lu et al., 2024).

PD patients exhibited significantly poorer quality of life and greater fatigue than controls, with higher PDQ-39 and PFS-16 scores across all domains. These findings confirm broad impairments in mobility, cognition, and emotional well-being, consistent with previous reports (Li et al., 2016; Hattori et al., 2022). The clinical burden observed may be mechanistically linked to dysregulation of the SIRT1/Nrf2/NLRP3 axis. Reduced SIRT1 and Nrf2 levels likely weaken antioxidant defenses, increasing oxidative stress that contributes to fatigue and cognitive decline (Franzoni et al., 2021). Conversely, activation of the NLRP3 inflammasome contributes to sustained neuroinflammation, which may be associated with non-motor symptoms such as mood disturbances in Parkinson’s disease (Zhang et al., 2015). Together, these findings suggest that oxidative stress and inflammation act synergistically to impair both molecular and clinical outcomes in PD, highlighting the therapeutic potential of targeting the SIRT1/Nrf2/NLRP3 pathway to improve quality of life and alleviate fatigue.

Correlation analyses in this study revealed strong links between molecular dysregulation and clinical severity in PD. Lower serum SIRT1 and Nrf2 levels were significantly associated with higher PDQ-39 and PFS-16 scores, indicating that reduced neuroprotection and antioxidant capacity contribute to poorer quality of life and greater fatigue. Both markers also declined with disease duration, reflecting progressive molecular deterioration. These results were in line with previous research (Zhu et al., 2021). Conversely, elevated NLRP3 levels correlated positively with PDQ-39 scores and disease duration, suggesting that heightened inflammasome activity parallels worsening symptoms and longer disease course (Soraci et al., 2023). Inter-marker relationships further supported this pattern—SIRT1 correlated positively with Nrf2 and inversely with NLRP3, while Nrf2 also showed a negative association with NLRP3—indicating that the balance between neuroprotective, antioxidant, and inflammatory pathways plays a central role in PD symptom burden beyond motor dysfunction.

Together, these findings suggest a tightly interconnected network in which decreased SIRT1 and Nrf2 compromise cellular defense mechanisms, facilitating inflammasome activation (Lu et al., 2024), which in turn exacerbates fatigue, diminishes quality of life, and correlates with disease progression (Batiha et al., 2023). These correlations provide both mechanistic insight and potential biomarker utility, highlighting that modulation of the SIRT1/Nrf2/NLRP3 axis could represent a promising therapeutic approach to alleviate both molecular dysfunction and clinical burden in PD.

The multiple linear regression analyses provide strong evidence that serum biomarkers of neuroprotection and inflammation, together with disease duration, are independent determinants of both health-related quality of life (HRQoL) and fatigue severity in PD patients. In the model predicting PDQ-39 total scores, SIRT1 and Nrf2 were independently associated with better quality of life, while NLRP3 and disease duration were independently linked to worse HRQoL. Similarly, for fatigue severity, higher levels of SIRT1 and Nrf2 predicted lower fatigue scores, whereas elevated NLRP3 and longer disease duration were associated with more severe fatigue. Notably, age, sex, and disease severity staging did not independently influence either HRQoL or fatigue, highlighting the central role of molecular alterations over demographic or clinical variables in determining patient-reported outcomes.

These findings underscore the mechanistic and clinical relevance of the SIRT1/Nrf2/NLRP3 axis. Higher SIRT1 and Nrf2 levels likely enhance neuroprotection and antioxidant capacity, mitigating oxidative stress and neuronal dysfunction, which in turn preserves energy, motivation, and functional abilities (Zamanian et al., 2023; Mayer et al., 2024). Conversely, elevated NLRP3 reflects systemic and neuroinflammation, which may exacerbate fatigue, cognitive impairment, and emotional distress, thereby reducing overall quality of life (Zhao et al., 2021). The independent associations of disease duration with both outcomes further suggest that progressive molecular dysregulation accumulates over the course of PD, amplifying clinical symptom burden.

The robustness of these findings was reinforced by multiple comparison corrections. After applying both Bonferroni and Benjamini–Hochberg false discovery rate adjustments, the majority of comparisons, including SIRT1, Nrf2, mobility, activities of daily living, stigma, and physical fatigue, remained statistically significant. Although NLRP3 lost significance with the stringent Bonferroni correction, it retained significance using the FDR method, indicating that the observed associations are not likely due to type I error and are consistent across multiple statistical approaches.

Receiver operating characteristic (ROC) curve analyses demonstrated that serum biomarkers SIRT1, Nrf2, and NLRP3 possess strong discriminative ability for distinguishing PD patients from healthy controls. These results are in line with previous reports (Zhu et al., 2021; Li et al., 2023; von Otter et al., 2014). SIRT1 exhibited excellent diagnostic performance with higher AUC and an optimal cutoff achieved both high sensitivity and specificity. Nrf2 also demonstrated high accuracy, with an AUC and a cutoff providing higher sensitivity and higher specificity. NLRP3 showed good discriminative performance, with 80.0% sensitivity and perfect specificity.

Overall, these findings indicate that dysregulation of neuroprotective and inflammatory pathways in PD has both biological and clinical significance. Reduced SIRT1 and Nrf2 levels, alongside elevated NLRP3, reflect impaired antioxidant defense and enhanced inflammation, correlating with greater fatigue and poorer quality of life. These alterations highlight the translational potential of the SIRT1/Nrf2/NLRP3 axis as a minimally invasive biomarker panel linking molecular dysfunction to patient outcomes. From a pharmacotherapeutic perspective, targeting this axis in randomized clinical trials offers novel opportunities to modulate disease mechanisms and improve clinical outcomes. Enhancing SIRT1 activity through agents such as resveratrol or metformin may support mitochondrial function and reduce oxidative stress, while Nrf2 activation could strengthen antioxidant defenses and protect against neuronal damage (Zamanian et al., 2023; Lu et al., 2016). Conversely, inhibiting NLRP3 inflammasome activation via selective inhibitors, IL-1β antagonists, or anti-inflammatory nutraceuticals may attenuate neuroinflammation and ameliorate both motor and non-motor symptoms. Collectively, these strategies underscore the potential of modulating the SIRT1/Nrf2/NLRP3 axis to achieve dual therapeutic benefits—slowing disease progression while improving quality of life in PD patients.

Because the present study is cross-sectional, it cannot capture the temporal dynamics of SIRT1, Nrf2, and NLRP3 alterations. Longitudinal studies are needed to determine whether these biomarkers exhibit stable trajectories over time, track disease progression, or change in response to therapeutic interventions. Such longitudinal validation would help establish their suitability for monitoring redox–inflammatory status and predicting clinical outcomes in PD.

This study has several notable strengths. First, it comprehensively integrates molecular, clinical, and statistical analyses, combining serum biomarkers (SIRT1, Nrf2, NLRP3) with validated patient-reported outcomes, including PDQ-39 for health-related quality of life and PFS-16 for fatigue severity. This multi-dimensional approach allows for a mechanistic understanding of how molecular alterations translate into clinically relevant symptoms in PD.

Second, the study employed age- and sex-matched healthy controls, minimizing confounding by demographic variables and strengthening the validity of between-group comparisons. The use of robust statistical methods—including Spearman correlations, multiple linear regression, and ROC curve analyses—enhanced the rigor of the findings, allowing identification of independent predictors of clinical outcomes and the diagnostic performance of biomarkers.

Third, multiple comparison corrections using both Bonferroni and Benjamini–Hochberg false discovery rate (FDR) adjustments were applied, ensuring that the results are robust and minimizing the likelihood of type I error. This methodological rigor strengthens confidence in the observed associations between biomarkers, fatigue, quality of life, and disease characteristics.

Finally, the study provides clinically relevant insights by linking serum biomarkers to patient-centered outcomes, emphasizing translational potential. The identification of SIRT1, Nrf2, and NLRP3 as independent predictors of both HRQoL and fatigue severity underscores their utility not only as mechanistic markers but also as potential targets for therapeutic intervention or disease monitoring.

Despite its strengths, this study has several limitations. First, Although the sample size (*n* = 30 per group) was determined *a priori* using a power calculation, we recognize that this relatively small, single-center cohort limits the generalizability of our findings. Effect-size estimates and biomarker associations may vary in larger or more diverse populations. Therefore, validation in larger, multicenter, and clinically heterogeneous cohorts will be essential to confirm the robustness, reproducibility, and broader applicability of the identified biomarker–clinical relationships.

Second, the study design was cross-sectional, which precludes establishing causality between biomarker alterations and clinical outcomes. Longitudinal studies are needed to determine whether changes in SIRT1, Nrf2, or NLRP3 predict progression of fatigue, quality of life decline, or disease severity over time.

Third, while serum biomarkers provide a minimally invasive measure, they may not fully reflect central nervous system processes, including local oxidative stress, neuroinflammation, or SIRT1/Nrf2 activity within dopaminergic neurons. Future studies incorporating cerebrospinal fluid (CSF) or neuroimaging markers could provide more direct insights into brain-specific pathological mechanisms.

Fourth, potential confounding factors such as comorbidities, medications, lifestyle factors, and nutritional status were not exhaustively controlled for, which may influence biomarker levels and clinical outcomes. Although age and sex were matched, other unmeasured variables could contribute to the observed associations.

Finally, the study did not evaluate the potential effects of PD subtypes or motor phenotypes on biomarkers and clinical outcomes, which may have distinct pathophysiological profiles. Stratified analyses in larger cohorts would help elucidate whether these findings are consistent across different PD subpopulations. Furthermore, the lack of direct mitochondrial functional assays. Although our biomarker and clinical data strongly implicate oxidative stress and inflammasome activation in PD, future work will include Seahorse XF extracellular flux analyses in patient-derived cells to quantify OCR/ECAR parameters (basal/ATP-linked respiration, maximal respiration, spare capacity) and test their associations with SIRT1/Nrf2/NLRP3 and clinical outcomes. This addition will more firmly anchor the mechanistic inferences to mitochondrial dysfunction, a recognized hallmark of PD.

## 5 Conclusion

In summary, PD is characterized by a significant reduction in neuroprotective and antioxidant biomarkers (SIRT1 and Nrf2) alongside elevated inflammatory activity (NLRP3), which collectively contribute to fatigue, diminished activities of daily living, and impaired health-related quality of life. These molecular alterations are closely interrelated, with SIRT1 and Nrf2 inversely associated with NLRP3, highlighting a mechanistic link between impaired neuroprotection, oxidative stress, and systemic inflammation. Serum levels of SIRT1, Nrf2, and NLRP3 independently predict patient-reported outcomes and demonstrate strong discriminative ability for distinguishing PD from healthy controls, underscoring their potential as minimally invasive biomarkers for diagnosis, disease monitoring, and therapeutic targeting.

Our findings emphasize the importance of integrating molecular and clinical assessments in PD and suggest that interventions aimed at enhancing SIRT1/Nrf2-mediated neuroprotection or reducing NLRP3-mediated inflammation may improve both symptom burden and quality of life. Future longitudinal studies with larger cohorts are warranted to validate these biomarkers and explore their utility in guiding personalized treatment strategies for PD patients.

## Data Availability

The raw data supporting the conclusions of this article will be made available by the authors, without undue reservation.
