# Supplementary material for: Interplay of oxidative stress and Inflammasome activation and clinical indices in Parkinson’s disease: insights from serum SIRT1, Nrf2, and NLRP3 levels and PDQ-39—a cross-sectional study
Source: Front Neurosci. 2026 Jan 12;19:1738871. doi: 10.3389/fnins.2025.1738871 (PMC12833396; doi:10.3389/fnins.2025.1738871)
Supplement: Supplementary file 1 [file Data_Sheet_1.docx]

*Model performance and visualization.* A penalized multivariable model including SIRT1, Nrf2, NLRP3, disease duration (PD only), and group (PD vs HC) yielded strong agreement between predicted and observed PSF‑16 scores (R² = 0.86; MAE = 0.30; **Figure S1**). The scatter illustrates that the model captures both the group‑level separation and within‑group variability in fatigue severity.


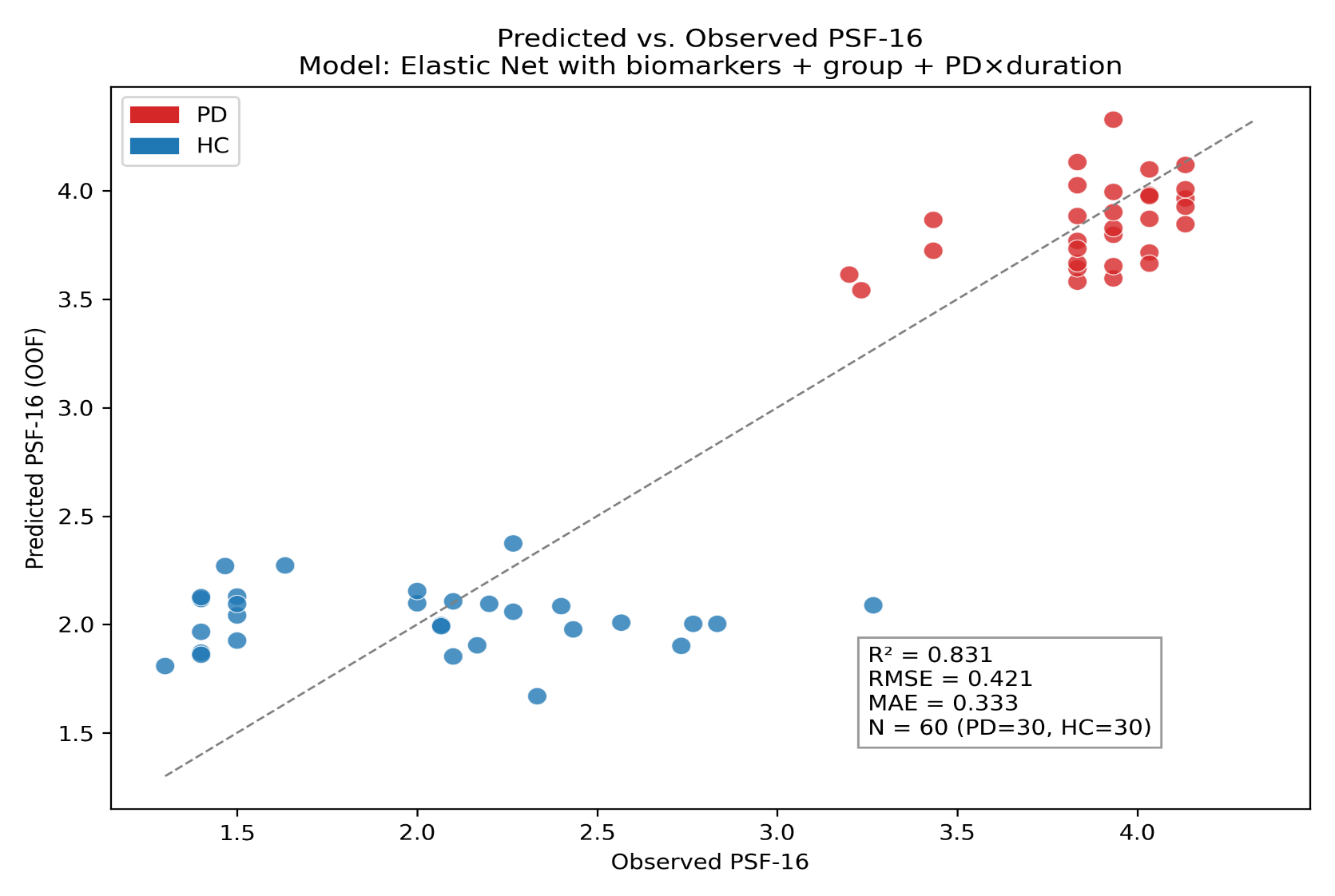


**Figure S1.** Predicted versus observed Parkinson’s Fatigue Scale‑16 (PSF‑16) scores from the multivariable model including SIRT1, Nrf2, NLRP3, disease duration (PD only), and group (PD vs HC). Points are colored by group (PD = red; HC = blue). The dashed line denotes the line of identity (y = x). Model performance on the full dataset: R² = 0.86, mean absolute error (MAE) = 0.30 PSF‑16 units.

Predicted vs observed plots were generated from the final multivariable model. Partial (added‑variable) regression plots were produced by residualizing each predictor and PSF‑16 on the remaining covariates and plotting the residual–residual relationship, with a fitted slope (Figure S2).


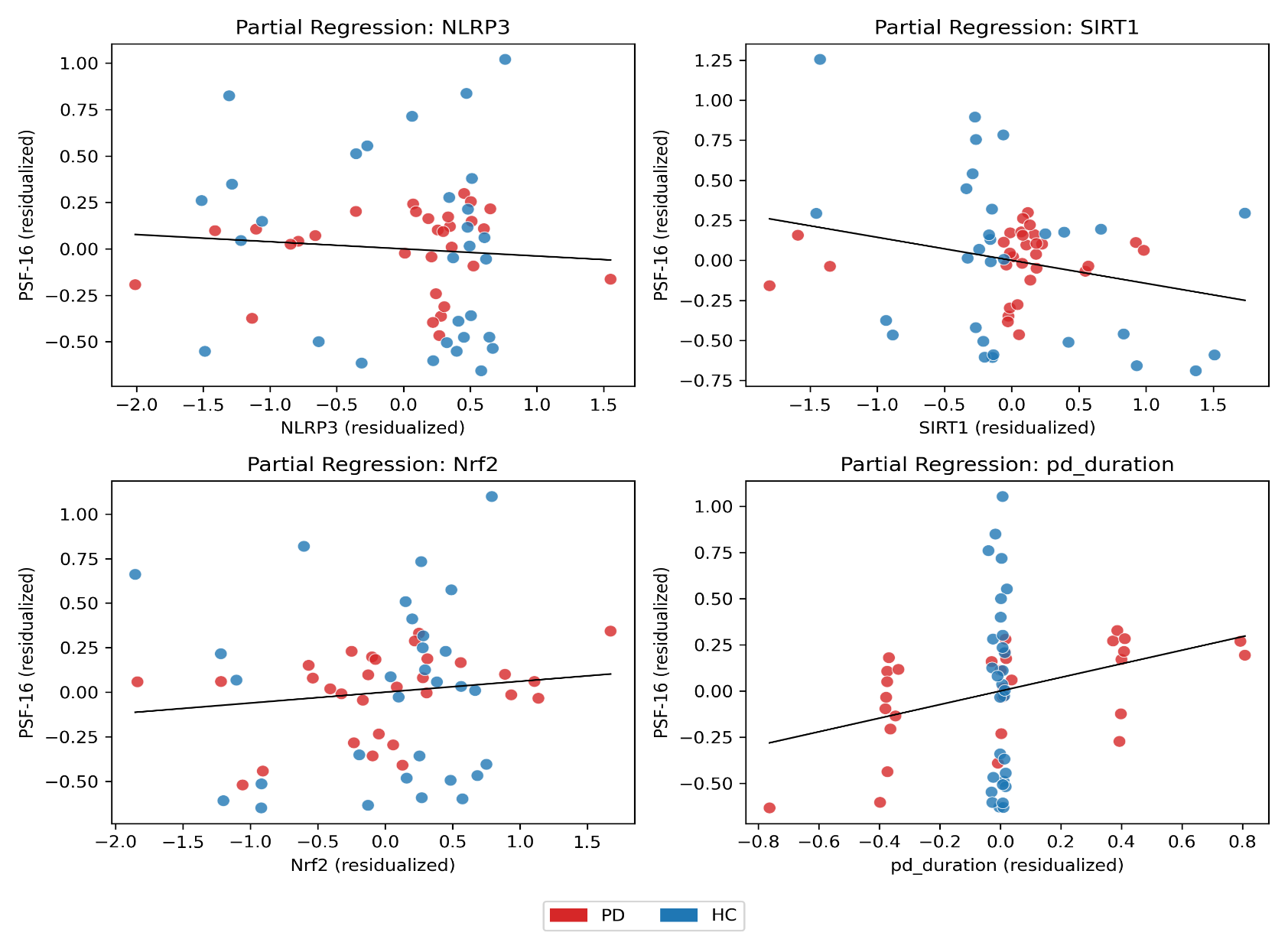


**Figure S2.** Partial regression (added‑variable) plots showing the relationship between (A) NLRP3, (B) SIRT1, (C) Nrf2, and (D) disease duration (PD only) with PSF‑16 after residualizing both the predictor and outcome on all other covariates in the model. Points are colored by group (PD = red; HC = blue). Solid lines indicate fitted slopes in the partial space.

Standardized coefficient plots display OLS coefficients computed after z‑scoring all predictors and the outcome; 95% confidence intervals were derived from the analytic OLS variance–covariance matrix (Figure S3).


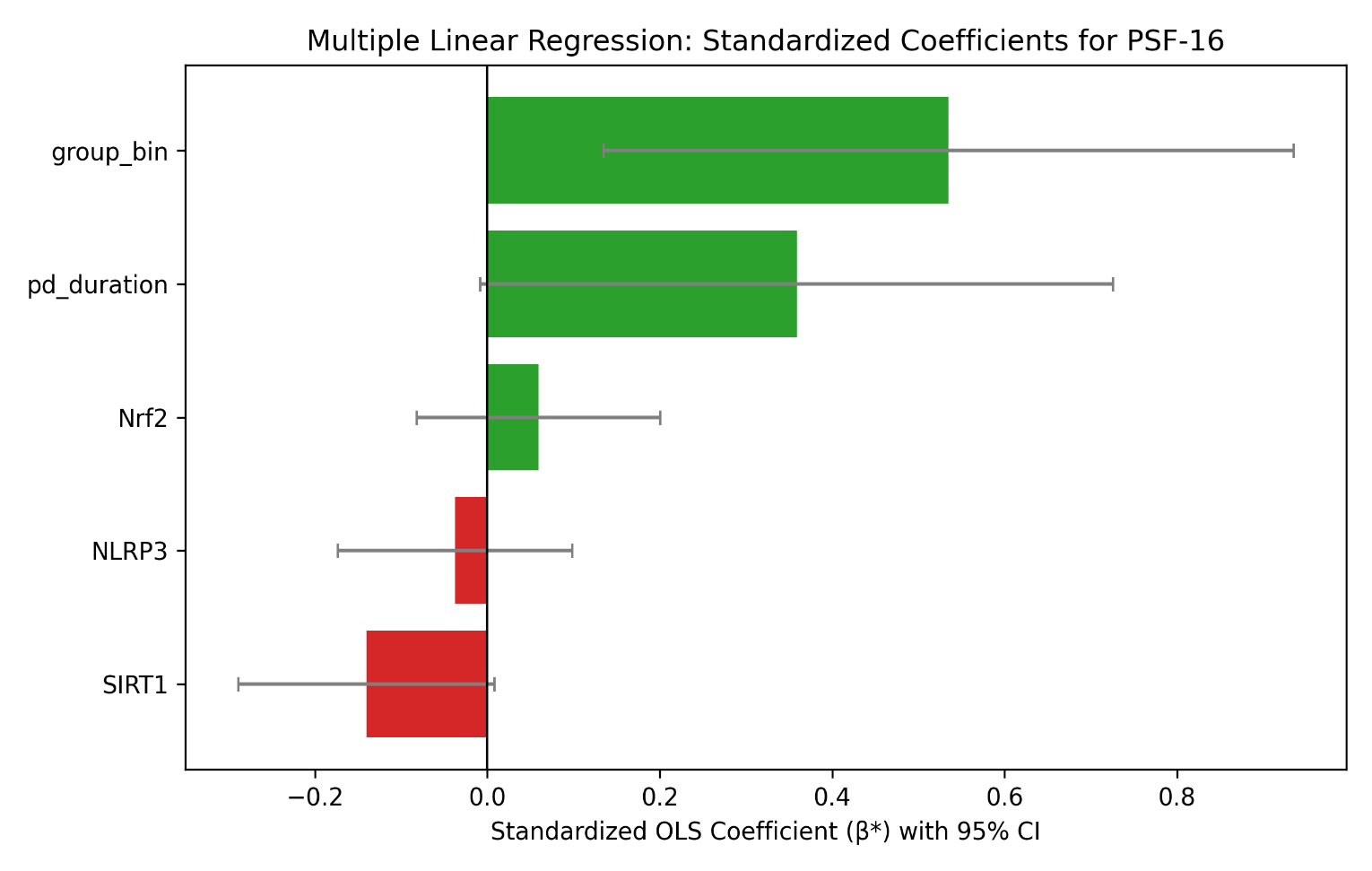


**Figure S3.** Standardized multiple linear regression coefficients (β*) for predictors of PSF‑16 with 95% confidence intervals. Positive bars indicate higher fatigue severity per 1 SD increase in the predictor; negative bars indicate lower severity. Predictors: NLRP3, SIRT1, Nrf2, PD vs HC (group), and PD disease duration.
